# Supplementary material for: Are carbon emissions trading and green financial instruments synergistic? -Comprehensive quantitative research based on content analysis
Source: PLoS One. 2024 Mar 7;19(3):e0298601. doi: 10.1371/journal.pone.0298601 (PMC10919601; doi:10.1371/journal.pone.0298601)
Supplement: S1 Data — (ZIP) [file pone.0298601.s001.zip › Data/地级市Z_F工作报告.zip!/╔╜╬≈/╦╖╓▌╩╨/└┤╫╘╣1⁄2╓┌║┼í╛┬φ┐╦╩2╛▌═°í┐_2003.docx]

各位代表：

　　现在，我代表市人民政府向大会作政府工作报告，请予审议，并请市政协委员和其他列席人员提出意见。

　　一、2002年政府工作回顾

　　一年来，市人民政府按照市委三届五次全会和市三届人大四次会议的部署，团结带领全市人民，紧紧围绕“创造特色，争先发展”的战略要求，以办好四十件实事为突破口，团结一致，扎实苦干，推动了各项工作的全面创新和各项事业的全面进步。

　　经济实力明显增强。国内生产总值突破百亿元大关，达到100．37亿元，增长13．5％，为1994年以来最好水平。财政总收入完成12．42亿元，增长23．15％；一般预算收入完成4.54亿元，增长28．84％，为历史上第三个高增长年。农业和农村经济全面发展。粮、油、菜产量在大面积退耕还林还草的情况下，仍保持正常年景的水平。畜牧业发展势头强劲。大牲畜、生猪、羊饲养量分别比上年增长6．1％、8．1％和9．7％，其中奶牛达到8．5万头，净增1万多头。工业增速加快，效益大幅提高。规模以上企业增加值增长14．8％，其中地方企业增长21．95％；规模以上企业实现利税增长39．22％，其中地方企业增长64．3％。煤、电、奶、瓷等主要行业全面增长，煤炭工业实现整体扭亏为盈。城乡居民收入增加。城镇居民人均可支配收入达到6304元，增长32．06％；农民人均纯收入2307元，增长55．4％。社会消费品零售总额、海关进出口总额等也都有较大幅度增加。

　　结构调整初见成效。在前两年成功起步的基础上，去年又启动了“2213”规划，实施了30个重点调产项目，其中13项建成投产，2项即将投产。三年累计实施重点调产项目46个，完成投资30多亿元。全市结构调整的成效已开始显现。以煤、电、奶、瓷为主导产业的格局正在形成。三年新增煤炭生产能力1700万吨，达到6000万吨；新增煤炭筛洗选能力2000万吨，达到5000万吨；新增发电量33亿千瓦时，达到142亿千瓦时；新增乳品生产能力9万吨，达到13万吨；新增日用瓷生产能力3亿件，达到4．3亿件。多成份的所有制格局和多渠道的投资格局正在形成。非国有经济比重由1999年的16.72％提高到50％，民营经济占到地方经济总量的65％以上。20多家国内外知名大企业大集团进入朔州，一批民营企业在结构调整中显示出主力军作用。

　　各项改革稳步推进。国企改革继续抓重点、破难点、全面推进，企业发展活力进一步增强。全市地方国有工业企业已改制115户，国有商贸流通企业已改制320户，分别占到企业总数的81％和96％。煤炭企业改革大面积推开。214座地方煤矿已改制186座，改制面达到87％。一批困难大企业的改革成果得到巩固扩大。王坪、小峪两大煤矿均实现了扭亏为盈。“两个确保”成效显著。13343名企业离退休人员按时足额领到了养老金，并一次性结清了4000多万元历史拖欠。符合保障条件的5993名下岗职工按时足额领到了基本生活费。城市低保工作基本实现应保尽保。

　　基础建设形成高潮。生态建设取得可喜成就。完成退耕还林24．8万亩、草地建设48万亩。大运高速公路绿化带、109国道退耕还林还草绿化带以及各县区的万亩生态园区等示范工程建设进展顺利。公路建设有了重大突破。大运高速公路朔州段及应县、怀仁两条连接线顺利通车，完成了朔蔚线改线和4公里世纪大道建设，新建了朔广线、应繁线等3条公路的出口路段。城市建设迈出新的步伐。东环路、开发路、建设北路、民福东街改造等工程如期竣工，城北万亩森林工程、崇福寺文脉工程和北关路改造工程完成预定任务，市二中、市三小和师范扩建工程全部建成，法院、检察院、交通局三座办公大楼和气象园区、体育广场均投入使用，市区到平鲁天然气长输管线基本建成，应县、山阴、怀仁天然气工程也已启动。各县区的城镇建设也都取得令人瞩目的成就。

　　社会事业全面发展。科技工作面向经济建设，组织实施市级以上科技项目48项，农业科技园区和企业技术创新基地建设有了新进展，科技成果转化步伐加快。教育事业达到一个新水平。“普九”成果扩大，“普九”人口覆盖率达到97％以上；高中教育发展迅速，质量提高。高中招生人数比上年增长19％，高考本科达线人数突破2000名。职业教育、成人教育都有较快发展。城乡卫生状况不断改善。人口过快增长势头有所遏制。环境达标成果得到巩固提升。民政、土地、宗教、人防、地震、气象、档案、老龄、残疾人等工作都取得新成绩。

　　精神文明和民主法制建设扎实推进。文明城市创建活动卓有成效，成为全省申报“全国文明城市”的三个先进城市之一。文化体育活动空前活跃，广大群众的精神生活更加丰富多彩。民主法制建设得到加强。市政府认真执行市人大及其常委会的决定和决议，自觉接受人大监督，并积极听取市政协、各民主党派、人民团体及各界群众的意见，办理人大代表意见、建议和政协委员提案共182件。政府职能进一步转变，行政审批制度改革有序推进，依法行政水平、行政效率不断提高。市政务大厅运转良好，得到群众肯定。“三务”公开不断拓展，全市及怀仁县推行政务公开的经验在全国、全省推广。严打整治斗争逐步深化，社会治安综合治理成效明显。廉政建设不断加强，消极腐败现象和行业不正之风得到遏制。

　　各位代表，这些成绩确实来之不易。这是市委坚强领导，市人大有效监督，市政协、各民主党派和各人民团体鼎力支持，全市人民共同奋斗的结果。在此，我代表市人民政府向全市人民致以崇高的敬意！向所有关心、支持朔州建设的驻朔单位及各级各界的同志们、朋友们表示衷心的感谢！

　　在肯定成绩的同时，我们也清醒地看到，在经济和社会生活中还存在许多不容忽视的问题。农民收入增长缓慢；结构调整任务仍很艰巨；财政收支矛盾仍很突出；环境污染问题仍很严重；投资环境不够宽松；社会治安状况群众不够满意；干部队伍作风建设仍需加强。对此，我们一定居安思危，高度重视，下决心克服和解决。

　　二、20O3年政府工作总的指导思想和主要目标任务

　　党的十六大提出了在本世纪头二十年全面建设小康社会的宏伟目标，为我们在新世纪新阶段继续前进指明了方向。全面建设小康社会，已成为全市经济社会发展的主旋律。市委三届七次全会描绘出今后一个时期我市全面建设小康社会的宏伟蓝图，明确提出要“团结奋斗五年，提前三年翻番，再造一个朔州”，就是在今后五年内，全市经济平均增长速度力求达到10.4％以上，到2007年国内生产总值达到167亿元，比2000年翻一番。这一目标是鼓舞人。心、催人奋进的、也是完全符合我市实际的。只要我们同心同德，埋头苦干，我们的目标一定能够实现。

　　按照这一宏伟目标，今年政府工作的总体要求是：以党的十六大精神和“三个代表”重要思想为指导，紧紧围绕全面建设小康社会的奋斗目标，坚持以经济建设为中心，以结构调整为主线，全面启动雁门关生态畜牧经济区和大运高速公路经济带建设两大战略，牢牢把握增加农民收入、增强经济实力、优化发展环境三个主攻方向，重点建设煤、电、奶、特（特色产业）四大支柱产业，促进全市经济持续快速健康发展和社会全面进步，为“提前三年翻番，再造一个朔州”而努力奋斗。

　　今年全市主要经济社会发展指标建议为：国内生产总值113亿元，增长10．5％；财政总收入13．96亿元，同口径增长12．4％；一般预算收入476亿元，同口径增长74％；全社会固定资产投资42亿元，增长10.5％；全部工业增加值503亿元，增长151％；社会消费品零售总额37．5亿元，增长 9．5％；海关进出口总额1900万美元，增长8％；城镇居民人均可支配收入6870元，增长9％；农民人均纯收入2600元，增长12．7％；城镇登记失业率控制在4．5％以内；人口自然增长率控制在千分之八以内。

　　为圆满完成上述指标，要着力抓好七个方面的工作：

　　（一）推进农业农村经济结构调整，努力增加农民收入

　　以促进农民增收为目的，以建设“生态畜牧经济区”为重点，大力推进结构调整，积极发展高效农业、特色农业、生态农业，进一步繁荣农业和农村经济。

　　以建设“畜牧强市”为目标，加快结构调整步伐，拓宽农民增收渠道。围绕市场调结构，立足特色调结构，瞄准绿色调结构，搞好服务调结构，逐步形成明显的产业优势和竞争优势。一是大力扩张畜牧业。坚定不移做生态文章，打乳业品牌，建畜牧强市，促农民增收。建设强大的奶源基地。制定实施全市乳业发展五年规划，指导乳业健康发展。大力发展规模养殖。全市培育百头以上奶牛养殖场100个，典型示范，整体带动，力争年底奶牛存栏达到10万头。加快建设肉牛肉羊基地。平川区要奶牛肉牛齐抓，山坡区要肉羊肉牛并举，今年力争出栏肉羊10O万只，肉牛饲养量有一个较大幅度的增长。二是不断优化种植业。从单纯注重品种调整，转到品种、品质调整并重，实施区域化布局、基地化生产，走出一条富有特色的种植业发展之路，创出一批“拳头”产品、品牌产品和绿色产品，提高市场竞争力和占有率。重点实施“双百万”工程，即以应县玉雄玉米深加工项目为龙头，在平川区建设100万亩优质高蛋白玉米基地；以朔城区嘉利土豆加工项目为龙头，在山区建设1O0万亩优质高淀粉马铃薯基地。三是积极发展加工业。着力培育一批带动力强、关联度高、辐射面广的龙头企业，一头连农户，一头通市场，带动农民致富奔小康。要“扶大龙”，重点扶持古城、雁门、康喜、田仁四大乳品企业的新项目建设，促进其进一步做强做大。要“聚小龙”，引导各类加工企业通过股份合作、引资嫁接、兼并收购等方式，进一步扩展规模。要“育新龙”，鼓励工商企业和社会各界投资兴办加工业，培育新亮点。四是强化引导和服务。牢固树立“扶持产业化就是扶持农业、扶持龙头企业就是扶持农民”的观念，制定实施优惠政策，支持产业化经营和发展。各级财政要拿出专项资金，吸引社会资金，重点投入奶源基地建设。金融部门给予信贷支持，尤其是小额贷款发放要向奶牛养殖倾斜，采用“龙头企业担保、承贷到户、奶款还贷”的方式，为养殖户提供发展资金。对规模养殖还要给予土地及其它方面的优惠。对骨干龙头企业的技改项目实行财政贴息补助，并在用地、信贷、税费等方面给予倾斜。对重点农村行业协会，市财政安排专项资金给予经费补助。对专业合作经济组织的经营收入，给予税收优惠。建立健全农业信息服务体系、动植物疫病防治体系、农畜产品质量监测检验体系、技术保障体系和良种繁育体系。市里组织建设三大基地，即以红旗牧场为主的科技推广试验基地、以农校为主的科技培训基地和以兴隆养殖场为主的胚胎移植基地。

　　以创建“绿色朔州”为龙头，加强生态水利建设，夯实农业发展基础。加大生态建设力度。继续搞好京津风沙源治理、首都水资源可持续利用及退耕还林还草三大工程，重点实施“001”生态建设规划，即涉及朔城区、平鲁、右玉的西部山区生态建设环形线和涉及朔城区、山阴、怀仁、应县的东部平川区生态建设环形线，以及大运高速公路两侧的生态建设工程。今年完成120万亩造林任务，其中退耕还林43．4万亩；完成人工种草100万亩。高标准建设“一条绿带、十颗明珠”。一条绿带，就是大运高速公路绿化带，两侧各100米范围内适宜退出的土地要全部退耕造林还草；十颗明珠，就是市和高速公路沿线四县区每家都要建设一个退耕还林生态示范区和一个龙头企业牵头的畜牧经济示范区。平鲁区和右玉县重点抓好国道两侧的退耕还林还草和小流域综合治理等生态示范项目建设。确保政策兑现。把国家给退耕户提供的各项优惠政策不折不扣地落实下去。创新组织形式。以明晰林权为核心，广泛吸引社会力量参与生态建设。注重工程效益。坚持造管并重，有计划地封山禁牧，努力提高成活率和保存率。着力建设节水朔州。加大对基本农田的保护和建设力度，搞好高效节水农田建设。以桑干河灌区为重点，在7大河灌区和4大井灌区建设100万亩高效节水农田。干旱地区发展小型、微型水利工程，充分利用有限的水资源。继续抓好农村饮水解困工程，力争年内再解决9万人的饮水困难和12万人的砷氟改水。

　　以实现共同富裕为宗旨，小康与扶贫两手抓，整体提高农民生活水平。制定实施新一轮小康建设规划，动员全民力量，掀起小康建设的新高潮，力争年内再有2个乡、73个村、1．3万人加入小康行列，有4个乡、97个村、8．3万人达到富裕型小康。坚持典型引路、整体推进。每个县区都要高标准建设几个示范点，带动农村小康建设整体上水平，以新的风貌迎接全省农村小康建设现场会在我市召开。切实抓好扶贫攻坚，全面完成9000人的扶贫移民任务，做好1800人的生态移民工作。用好移民资金，抓好产业开发，积极探索、正确引导移民解决生产出路和生活致富问题。

　　以推进体制创新为重点，继续深化农村改革，进一步激活农村经济。在稳定农村基本政策和家庭承包经营制的前提下，按照“依法、自愿、有偿”的原则，适时进行土地承包经营权合理流转，发展规模经营。继续完善“四荒”和小型水利工程拍卖治理机制，引导和动员全社会力量，承包、购买荒山荒坡进行治理。全面推进农村税费改革，确保农民负担减轻、农村义务教育经费落实和农村基层组织正常运转。深化粮食流通体制改革，切实保护农民利益。大力推动农村经营体制创新，积极发展专业合作组织和行业协会，办好奶业协会，培育壮大经纪人队伍，带动农民走向市场。

　　（二）全面提升工业发展水平，增强总体经济实力

　　坚定不移实施“工业强市”战略，坚持不懈推进结构调整，走新型工业化道路，推动全市工业向着规模扩张、结构优化、产业升级和效益提高的方向迈进。

　　依托开放引进，带动经济结构更快调整。紧紧抓住我国经济持续快速增长的有利时机，以更大的力度、更快的步伐，努力推动全市工业结构在更大范围、更深层次上实现调整和优化。在调整方向上，立足资源型城市的实际，走可持续发展之路，推进资源优势向经济优势转化，着力做好两个方面的工作：一是继续做大做强传统产业，加快“煤都”、“电都”建设。认真抓好一批大型矿井的建设和中小型煤矿的改造，促进煤炭生产的规模化经营；继续做好煤炭运销工作，提高洗选比例和煤炭质量，保持和扩大既有的信誉与市场；高度重视煤炭加工转化工作，积极兴办洗精煤、水煤浆和矸石电厂等项目，延伸产业链条；支持国家大型电力项目建设，大力发展高载能工业，逐步形成煤、电、高载能一体化产业。二是培育壮大新兴产业，增强后续替补能力。加快乳品、陶瓷、化工等多元主导产业发展。特别是要紧紧抓住乳品、陶瓷两个特色优势产业，坚持以项目建设为核心，进一步加强组织领导，加大推动力度，抓好资金、技术、质量等关键性问题，加快在建项目建设和建成项目的达产达效，逐步建成在全省乃至全国有重要影响的“奶都”、“瓷都”，实现由能源工业城市向特色工业城市的转变。在依托力量上，走好开放引进、联大联强、联新联优的路子。牢固确立实施大开放促进大引进、凭借大引进促进大发展的观念，全力推进体制机制的创新，全面推进软硬环境的优化，创造一切有利条件，提供一切可能的优惠，建立对大企业的保护机制，促成更多的大企业、大集团向朔州集聚和发展，进一步扩大其投资规模。今年在继续抓好大唐公司2X50万千瓦机组建设的同时，开工建设21个项目，包括神华集团朔西1000万吨煤矿、陶村300－50O万吨煤矿、木瓜界集运站200万吨洗煤厂建设和木瓜界煤矿改造，露矿500万吨矿井、2X5万千瓦矸石电厂建设以及安格公司高岭土项目二期扩建、狮头集团35万吨水泥生产线、省煤炭进出口公司东升煤矿100万吨矿井改造、同煤集团420万吨矿井改造、晋能集团2．5万吨铝硅合金、古城集团8条液态奶生产线、雁门乳业公司4条液态奶生产线、康喜集团2条液态奶生产线、汇源应县分公司6条液态奶生产线、玉雄公司1O万吨玉米淀粉深加工、嘉利公司20万吨马铃薯综合加工、汾酒集团8万吨啤酒改造一期工程、皇威公司64万吨硅铁合金等项目。其中露矿500万吨矿井、安格公司二期扩建、晋能2．5万吨铝硅合金、皇威64万吨硅铁合金4个项目力争当年完成。抓好金海洋300万吨洗煤厂、小峪煤矿9O万吨洗煤厂等项目的投产达效。做好大唐2X60万千瓦机组、中电集团投资公司2X60万千瓦机组、露矿2X13．5万千瓦机组以及中煤、同煤、鲁能集团等在湖南、朔西煤田开发项目的前期准备工作。

　　依托园区建设，推动民营经济更大发展。以骨干企业和拳头产品为依托，以城镇和交通要道为重点区域，按照布局集中化、经营规模化、生产现代化和产权多元化原则，全面规划、分步实施，逐步建设一批布局合理、设施配套、管理先进、环境优美、特色鲜明、实力雄厚的经济园区，为全市民营经济更大发展搭建平台。重点抓好“两带七区”建设，即神头到平鲁一级公路经济带，包括市经济技术开发区、神头工业园区、平鲁工业园区，与安太堡、安家岭两大矿区及神头两大电厂连成一片，形成以重工业和高新技术产业为主导的经济走廊；大运高速公路经济带，包括怀仁云东开发区、嘉明工业园区、山阴古城工业园区、应县城东工业园区，与广武生态旅游区相连，形成以轻工业和旅游业为主导的经济走廊。同时支持右玉搞好威远工业园区建设。力争通过几年努力，由分布在两条交通大动脉上的园区聚起一批富有实力、充满活力的企业集群，进而形成“人字形”经济隆起带，带动两翼，辐射周边。科学规划，高标准设计，努力营造高品位、有活力的投资环境和园林式的生态环境。积极筹措资金，搞好园区基础设施建设，引导金融、保险、邮电等服务部门在园区安家落户，完善园区服务功能。立足当地优势，根据各自条件，确定发展战略和突破口，力求做到一园一品，形成特色。着眼长远，把好入园关口，技术含量上要有高标准，土地使用上给优惠，政策服务上给倾斜，增强吸引力和聚合力。通过园区的集群和带动，促进民营经济大发展。力争年内新上50万元以上的投资项目超过100个、营业额500万元以上的企业发展到50家、纳税500万元以上的企业达到20家。

　　依托改革创新，充分激发企业发展活力。全面推进企业组织结构、产权结构、经营机制等方面的创新，真正形成生机勃勃的市场主体。坚持民营化方向，加快国有企业的改制改组，年内达到地方“零国有独资企业”。深化煤炭企业改革，继续推进煤电联营、国地矿联营、产销联营和与大企业的联合协作。推动优势骨干企业建立现代企业制度，构建合理的法人治理结构。搞好市天然气公司的改制改组，引深王坪、小峪、煤运、矿业等大企业的改革，抓好朔芳亚麻公司、三元炭素公司、锅炉厂等困难企业的脱困工作。同时引导民营企业建立合理的劳动、分配和管理制度。

　　依托先进的技术与管理，实现经济质量和效益的进一步提高。始终把经济发展的质量放到突出的位置，把效益作为最终的衡量标准，努力走出一条速度、结构、效益相统一的新型工业化路子。引进项目坚持高标准，新建项目坚持高起点，坚决杜绝浪费资源、污染环境的新项目建设。积极采取新技术改造提升传统产业，大力发展高新产业项目。把信息化作为工业化进程的先导环节来抓，推进信息技术在生产领域的广泛应用，加速经济管理的信息化进程。完善金税、金财、金卡工程，实施煤炭税费的电子化征缴，推进“企业上网工程”，启动电子政务工程，逐步实现信息资源共享。

　　（三）进一步扩大社会需求，强化财税金融工作

　　扩大投资和消费需求，大力开拓城乡市场。政府有关部门要抓住机遇，明确任务，积极向上争取投资，用好国家电力、水利、交通、生态、环保等专项资金；通过大开放、大引进，广泛吸引外来投资，激活民间投资，充分发挥投资对经济增长的拉动作用。积极开拓消费市场，引导城乡居民增加即期消费，推进信贷消费。深化住房货币化改革，培育住房二级市场，扩大住房消费。引深流通体制改革，积极发展连锁经营、配送经营、代理制等现代流通方式，进一步搞活商贸经济。继续整顿和规范市场经济秩序，抓好专项整治工作，深入开展“打假”活动，致力建设规范有序的市场环境。

　　千方百计增收节支，增强财政调控能力。努力壮大主体财源，积极开辟新的财源，充分利用贴息、融资等调控手段，支持经济发展，提高财政收入水平。坚持依法治税，强化征管，应收尽收。严格执行支出预算，依法保证重点支出，控制和压缩一般性支出，制止各种铺张浪费行为，确保财政收支基本平衡。深化财政体制改革，逐步建立公共财政框架。加快部门预算改革，推进国库集中收付制度，依法规范政府采购行为，引深收支两条线工作，强化预算外资金管理，加强财政性资金使用情况的监督检查和审计监督。按照中央和省的部署，逐步建立国有资产分级管理新体制。

　　加大信贷支持力度，切实改进金融服务。政府、企业与银行要建立新型的协商机制，经常互通情况，增进了解，促进合作。积极调整信贷结构，找准金融与经济共同发展的切入点，做好扶优、扶强、扶特工作，对重点调产项目、生态畜牧建设项目及基础设施建设项目，大力给予信贷支持，力求取得双赢效益。

　　着力建设信用朔州，营造良好的信用环境。各级各部门带头讲信用，带头建立信用承诺制度，带动和促进全社会信用建设。组织开展创建信用单位、信用乡镇和信用企业活动，积极支持金融部门创建金融安全区，严厉打击逃废银行债务行为。

　　（四）完善城市功能，加快城镇化进程

　　紧紧围绕建设“北方生态园林工业城市”，坚持硬件与软件齐抓，建设与管理并重，城市与城镇互动，推进城镇化进程，促进城乡协调发展。

　　提升城市建设与管理水平。完善城市规划。把规划作为先导性的工作来抓，依据城市特色定位，高标准完成城市总体规划的修编及相配套的专业规划、建制镇规划的编制。加强规划管理，充分发挥规划在指导城市建设中的龙头作用。加快城市建设。以新的理念和思路，实施经营城市战略，多途径经营城市，走“以城养城、以城兴城”之路。按照市场规则，盘活存量土地，靠土地生财聚财。引入竞争机制，建立城市资产评估制度、有偿出让制度和招投标制度，加快市政公用行业的市场化进程。对供水、供气、供热以及污水、垃圾处理等部分经营性市政设施，通过公开招标，拍卖经营权；对环卫、园林、路灯、道路等非经营性的市政设施，通过公开竞标，选择管护者。多渠道、多形式吸引市内外社会资金，建设和完善水、电、路、气、热、通讯和环境保护七大系统，进一步做大、做强、做美城市。今年集中抓好七里河二库建设及三库规划、天然气开发利用、污水处理厂建设、垃圾处理厂筹建、二水厂扩建、市电力园区和交通通信小区建设、双语学校和职业学校建设、汉墓博物馆完善配套、市妇儿活动中心筹建、崇福寺文脉工程和北关路改造续建、火车站站前路筹建，“城中村”改造前期准备等一批重点工程。搞好城市绿化。年内新增城市绿化面积45万平方米；确保城市绿化覆盖率增加2个百分点，达到27％；人均公共绿地增加1平方米，达到14．5平方米。重点推进城北万亩森林工程和七里河公园、开发路、建设路、民福东街、立交桥周边及机关庭院、居民小区的绿化。加强城市管理。“城市管理年”活动要搞得有声有色、富有成效。广泛开展“爱我朔州、建我家园、树我形象”活动，强化市民的城市意识、责任意识和参与意识，积极投身于文明城市创建。完善“两级政府、三级管理”的城市管理体制，强化城区、街道、社区的管理职能，并借鉴兄弟城市经验，建立市区统一的综合执法体系，相对集中行政处罚权，严格规范执法，提高执法效率。建立健全管理制度，改革城市管理方式，建立长效管理机制，加强执法队伍建设，落实严管重罚措施，集中解决城市的脏、乱、差问题。社区建设要摆上议程，点上示范，面上拓展，逐步实现就业和社会保障到社区、城市管理到社区、综合治理到社区、家政服务到社区。今年重点抓好市委家属小区、市府家属小区、府南小区、七里河小区、民福小区五个示范区建设。

　　加快小城镇建设步伐。县城作为小城镇建设的重点，要加大建设力度，集中抓好基础设施的配套完善和旧城改造，其它建制镇建设全面起步。吸引多方投入，完善服务设施。加快户籍制度改革，为农民进城务工经商创造条件。清理和纠正歧视性政策和乱收费，保护进城农民的合法权益。进一步促进产业集聚和人口集聚，提高小城镇承载力。

　　大力发展交通事业。重点搞好井坪至神头电厂、世纪大道南北延伸和怀仁、应县两条高速公路连接线拓宽改造四条一级公路建设，改建109国道15公里、朔平线5公里、右玉通市油路胡村至吴马营段、平万线平鲁至偏关界、怀仁陈家堡至浑源界五条二级公路，新改建山阴至和林格尔、南榆林至广武、朔州至神池、应县至繁峙四条出境公路，完成右玉李达窑乡通油路、市区至沙得河、应县金城镇至下马峪三条三级公路和5O个村通油路，开工建设市物流中心、市客运北站和怀仁、应县、右玉三个汽车站。

　　（五）做好就业再就业工作，健全社会保障体系

　　真正带着感情，带着责任，落实各项救助措施，认真解决弱势群体的基本生活问题。

　　努力扩大就业。把改善就业环境、增加就业岗位作为政府的重要职责，纳入经济和社会发展计划。广开就业门路，大力发展非公有制经济、中小企业、劳动密集型产业和第三产业，积极开发社区服务业，扩大劳务输出，拓宽就业渠道，千方百计促进就业再就业。规范发展劳动力市场和人才市场。加大投入，完善设施，建立信息网络，强化就业指导和服务，积极倡导和推广灵活多样的就业方式。多形式开展培训工作。今年新就业人员要全面培训，下岗职工培训率要达到70％以上，并实行就业和职业资格证书准入制度。实施再就业援助。制定和落实各项扶持政策，将“4050”（女40岁、男50岁以上）再就业困难职工作为主要援助对象，政府投资开发各类公益性岗位，提供多种再就业帮助。今年下岗职工再就业率争取达到40％以上。

　　加快社会保障体系建设。完善下岗职工基本生活保障制度，确保下岗职工基本生活费按时足额发放。妥善解决下岗职工出“中心”中遇到的困难和问题。完善城镇养老、失业保险和医疗保障制度，努力扩大覆盖，强化基金征缴，推进社会化管理，加快非公有制企业和机关事业单位参加社会保险的进程。完善城市居民最低生活保障制度，切实解决城市特困家庭的生计问题。积极探索建立农村养老、医疗和最低生活保障制度。

　　深化分配制度改革。完善工资专户制度，落实中央即将出台的增资政策，确保公教人员工资按时足额发放。各类企业都要建立完善用工制度和分配制度，强化激励机制，实行劳动、资本、技术和管理等生产要素按贡献参与分配，并严格执行国家有关规定，依法缴纳各种社会保险，落实最低工资标准。

　　（六）加强精神文明建设，全面发展各项社会事业

　　高度重视精神文明建设，认真实施科教兴市战略，努力实现经济与社会协调发展。

　　加强思想道德建设。强化思想政治教育，坚持用“三个代表”重要思想武装干部，教育群众，凝聚全市人民的力量。认真贯彻《公民道德建设实施纲要》，加强社会公德、职业道德和家庭美德教育，引导全市人民树立热爱朔州、建设朔州、发展朔州的共同信念。以建设文明城市为重点，深入开展群众性精神文明创建活动，努力形成良好的城市形象。

　　加快科技进步和技术创新。围绕结构调整这一主线，找准切入点，集中推进科技兴农、科技兴企两大工程，提升主导产业的科技含量。开放式地推进科技进步与技术创新，壮大科技队伍，坚持产学研结合，引进技术与引进项目、人才、资金结合，采取委托开发、联合开发、购买专利、聘请人才、技术入股、技术承包等多种形式，加快新技术的推广和新成果的转化，重点在煤炭深加工、高载能工业、乳制品业及种养业上突破。

　　大力发展教育事业。认真落实教育优先发展的战略地位，积极推进教育创新，发展素质教育。巩固扩大“两基”成果，切实办好义务教育。推进教育扶贫工作，多渠道增加教育投入，基本完成中小学危房改造任务。采取行之有效的政策措施，稳定提高农村教师队伍素质，提高农村义务教育质量，积极扩大城市小学、初中的办学规模，统筹解决城市学校班容量过大的问题。在年内基本完成右玉县“普九”工作，全面提升其余五县区“普九”水平。整合教育资源，扩大优质高中教育。通过新办和扩建学校，逐步解决示范中学的初、高中分离。市一中、二中和县区重点中学要扩大高中招生规模。大力发展职业教育、成人教育，积极发展现代远程开放教育。办好师范学校，做好升格为专科学校的各项准备工作。深化教育改革，继续推行中小学校长负责制、教职工会员聘用制和结构工资制，增强办学活力。全面贯彻国家《民办教育法》，鼓励和规范社会力量办学，形成公办学校与民办学校相互促进、共同发展的格局。

　　积极发展文化、卫生、体育等事业。培育富有地方特色和鲜明时代特征的朔州文化，努力建设文化强市。重视基层文化队伍建设，经常组织开展群众性的文化活动，活跃人民群众的精神生活。落实文化产业政策，建立投入保障机制。搞好文化基础设施建设，加快有线电视网络改造与整合步伐，巩固提高广播电视“村村通”成果。加强重要文化遗产的保护与开发，积极打造以应县古塔、城区古寺、山阴广武古城为代表的朔州历史文化及旅游文化品牌。深化医疗卫生体制改革，完善市级医疗服务机构，抓好农村合作医疗试点，实施农民健康工程。规范医疗服务市场，加强食品药品监督检查，保障人民生命健康。发展竞技体育，开展全民健身活动。加强民兵预备役部队建设，增强全民国防观念。认真做好民政、防震减灾、气象监测及保密、档案、外事等工作。依法保护妇女、儿童、老年人和残疾人的正当权益。

　　做好计划生育和环境保护工作。全面贯彻国家《人口与计划生育法》和省《计划生育条例》，稳定低生育水平，完成人口控制目标。加大对重点污染区域、重点污染源的整治力度，集中抓好城市和县城的大气污染治理及垃圾、污水治理，提高全市整体环境质量。依法保护和合理利用土地、矿产和水资源。

　　各位代表！维护社会稳定是保障经济社会全面发展的基本条件，也是政府工作的一项重要任务。高度重视、分级负责、认真做好信访工作，满腔热情地解决好群众关注的热点和难点问题，努力把不稳定因素消除在萌芽状态。引深严打整治斗争，依法打击各种犯罪活动，深入开展同“法轮功”等邪教组织的斗争。坚持打防并举、群防群治，落实社会治安综合治理各项措施，着力改善社会治安环境。把安全生产摆到更加突出的位置，进一步明确责任，超前防范，严格执法，坚决杜绝特大事故。

　　（七）加强政府自身建设，努力提高服务水平

　　搞好政府自身建设，加快职能转变，进一步提高工作质量，以良好的形象去凝聚群众，以模范的行动去感染群众，以扎实的工作去带动群众，向全市人民交出一份满意的答卷。

　　努力学习，与时俱进。面对新形势，完成新任务，我们必须学习、学习、再学习，实践、实践、再实践，既注重学习马列主义、毛泽东思想、邓小平理论和“三个代表”重要思想，又要努力学习经济、法律、科技、管理等知识，使思想水平、知识水平和实际工作能力适应时代前进的需要。建立制度，健全机制，营造氛围，创建学习型机关、学习型单位，创建学习型政府，推动学习型城市建设。紧扣加快发展这一主题，紧密结合朔州改革开放和经济建设的实践，紧密联系本地区、本部门的实际，做到勤于思考和研究问题，善于与时俱进提出新思路、新办法，不断把学到的东西运用于实践，把学习成果转化为推动朔州各项事业发展的智慧和能力。

　　深化改革，转变职能。继续推进行政管理体制改革和干部人事制度改革，全面推行事业单位聘用制度，逐步形成行为规范、运转协调、公正透明、廉洁高效的行政管理体制。加快政府职能转变，创新管理方式，进一步减少和规范行政审批；推进“阳光”行政，完善服务载体和制度，全力办好市政务大厅和县级便民服务中心。大力开展机关效能建设，建立抓落实的机制和制度。每项重点工作都要专人负责，明确目标、任务和责任，都要检查、督促、考核、奖惩到位。以人查事，以事查人，奖罚分明，公示于众。大兴求真务实之风，在鼓实劲、办实事、出实效上下功夫，反对形式主义、官僚主义，力戒说了不干、干而不实的不良作风，下决心整治“只要俸禄不要奉献”的庸俗风气，提高机关服务质量和效率，进一步促进软环境的改善。

　　以民为本，为民解忧。人民群众是我们的衣食父母，是我们克服困难、不断走向胜利的力量源泉。我们每一位政府工作人员都要时时刻刻牢记宗旨，把全心全意为人民服务作为政府一切工作的出发点和归宿点，体现在行动上，落实到实践中，做到经常察民情、听民声、解民忧，把民情动态作为第一信号，把群众意愿作为第一准则，把富裕人民、造福人民作为第一追求，真正做到“权为民所用，情为民所系，利为民所谋”，自觉地运用人民赋予的权力，为人民群众办实事，办好事，解难题，排忧愁，尤其要充分关注生活困难的群众衣食温饱和安危健康，确实做到一枝一叶总关情，不以善小而不为。

　　严格自律，依法行政。毫不放松地推进党风廉政建设。抓好领导干部的廉洁自律，落实党风廉政建设责任制，模范遵守廉政准则和各项制度，知廉耻、讲廉洁，既管好自己，又管好亲属和身边工作人员。大力查办各类违法违纪案件，集中突破一批有影响、有震动的大案要案，毫不手软地清除政府公务员中的腐败分子。大力纠正行业不正之风，认真解决群众反映强烈的问题。标本兼治，强化监督，注重从源头上预防和解决腐败问题。依法行政，依法办事。认真执行市人大及其常委会的决议和决定，坚持重大事项报告制度，自觉接受法律监督。密切与市政协和各民主党派、人民团体的联系，积极输市人大代表、政协委员的意观、建议和提案。建立政府重大决策听证制度和专家咨询制度，推进决策的民主化、科学化、法制化。建立健全行政执法责任制、执法过错追究制，强化行政复议、行政诉讼和行政执法的监督，依法规范政府行为，坚决做到有令必行、有禁必止、政令畅通。

　　各位代表，我们已站到了一个新的历史发展起点上。全面建设小康社会，实现朔州发展的新跨越，是全市人民的期盼，是时代赋予的神圣使命。让我们在中共朔州市委的坚强领导下，认真实践“三个代表”重要思想，务必保持谦虚谨慎、不骄不躁的作风，务必保持艰苦奋斗的作风，团结一致，埋头苦干，全力做好今年的工作，为创造我们的幸福生活和美好未来而努力奋斗！
